# The interRAI Child and Youth Suite of Mental Health Assessment Instruments: An Integrated Approach to Mental Health Service Delivery

**DOI:** 10.3389/fpsyt.2022.710569

**Published:** 2022-03-17

**Authors:** Shannon L. Stewart, Angela Celebre, Valbona Semovski, John P. Hirdes, Chris Vadeboncoeur, Jeffrey W. Poss

**Affiliations:** ^1^Faculty of Education, Western University, London, ON, Canada; ^2^Faculty of Applied Health Sciences, University of Waterloo, Waterloo, ON, Canada; ^3^Faculty of Medicine, University of Ottawa, Ottawa, ON, Canada

**Keywords:** children’s mental health, care planning, outcomes, quality indicators, case-mix systems, psychometric properties, service integration, interpersonal trauma

## Abstract

Various biological, social, psychological, and environmental factors impact children and youth living with mental health problems across their lifespan. To meet the wide-ranging challenges of mental illness, service system integration is needed to improve efficiencies and reduce fragmentation. Unfortunately, the mental health system has been plagued by the lack of coordination across services. There is a general consensus that mental health service delivery must ensure a child or youth’s needs are addressed in a collaborative, coordinated, and seamless manner. A key element to successful integration is the development of a comprehensive standardized screening and assessment system. Numerous assessments have been developed to assess child mental health and functioning, but they typically have a very narrow focus with limited use and utility. Not only does this reduce the ability to take a life course perspective to mental health, but this uncoordinated approach also results in redundancies in information collected, additional resources, and increased assessor burden for children, youth, and their families. The interRAI child and youth mental health assessment suite was developed in response to the need for an integrated mental health system for young persons. This suite includes screening and assessment instruments for in-patient and community settings, emergency departments, educational settings, and youth justice custodial facilities. The instruments form a mental health information system intentionally designed to work in an integrated fashion beginning in infancy, and incorporate key applications such as care planning, outcome measurement, resource allocation, and quality improvement. The design of these assessment tools and their psychometric properties are reviewed. Data is then presented using examples related to interpersonal trauma, illustrating the use and utility of the integrated suite, along with the various applications of these assessment systems.

## Introduction

Mental health and wellness begins in infancy, as the persistence, stability, and negative long-term consequences of early mental health problems are evident across the lifespan (1). Infants who experience serious adversity in the first few years of life, such as exposure to violence or trauma, are more likely to demonstrate emotional and behavioral problems (2). Young persons with mental health issues are at increased risk for poor academic achievement, underemployment, substance abuse, and impaired social and emotional functioning (3, 4). Additionally, childhood mental illness has been associated with suicide, homicide, criminal activity, as well as institutionalization and incarceration (5, 6). Despite approximately 10–20% of children and youth experiencing a mental health problem, the majority go undiagnosed and untreated (7, 8).

Untreated mental health problems in childhood can persist into adulthood with approximately 70% of adult mental health issues beginning in childhood and adolescence (9). While there has been a substantial amount of research examining the natural progression of child psychopathology, studies exploring the continuity of mental health symptoms across the lifespan are limited, often due to issues regarding appropriate measurement. Some researchers have found that while the overall prevalence of disorders may be comparable, the patterns of specific disorders vary based on age (10–12).

Theoretical frameworks to conceptualize children and youth mental health have been investigated over several decades. A comprehensive approach to understanding children’s mental health is appropriate considering the strengths and needs of young persons are shaped by interactions of intrapersonal and interpersonal factors over time (13). Examples include, but are not limited to, physical health, disability, social relationships with family and peers, and education. Mental health concerns in young persons are becoming increasingly prevalent with approximately 10% of those referred for services presenting with increasing complexity (14, 15). These children account for a significant and disproportionate amount of mental healthcare spending and often require ‘episodic, chronic, and ongoing care’ from multiple service sectors, including costly residential and in-patient services (12, 16–21).

Mental health disorders have a societal and economic impact, with direct and indirect costs exceeding $59 billion annually (22). Given the sequalae of untreated mental health problems, coupled with the corresponding economic costs, it is critical for children, youth, and their families to be identified early and have access to timely and integrated services (23, 24). To improve the responsivity of mental health services, it is imperative to adopt an orientation addressing all aspects implicit in shaping a child’s educational, physical, socio-emotional, and developmental well-being (13)^1^.

Early identification of mental health needs is essential, as it fosters early intervention and prevention, thereby reducing the need for intensive resources and crisis services (26, 27). Investments made before birth have a higher return, highlighting that prevention is more cost effective than later remediation (28, 29). Early identification and intervention have the potential to decrease impairment, diminish distress, and reduce the number of young persons who require treatment services later in life. As a result, these children and youth are afforded a greater opportunity to be productive contributors within their communities.

The mental health system has been plagued by high fragmentation and low funding, particularly in children’s mental health (26). At the jurisdictional level, mental health programs and services are typically delivered with little coordination among governmental agencies (8, 30). As the number of service providers involved increases, it becomes more difficult to coordinate services due to a lack of communication and integration (21). Tragically, these compounding factors create steep barriers for families to access appropriate mental health services (31). To alleviate some of the stress experienced by families navigating the system, the development of an integrated standardized mental health assessment system that uses a common language linking community agencies, hospitals, child welfare, youth justice facilities, and educational settings while providing opportunities for transitional care into adulthood is needed (32).

In response to the call for an effective mental health information system for adults, interRAI has developed a number of assessment and screening instruments [e.g., (33)]. For an in-depth review of the adult mental health suite of instruments, please see our previously published work (34). Despite advances within the adult system, children’s mental health has continued to lack a harmonized approach to service delivery, in part, due to the lack of psychometrically sound instruments spanning service sectors. While various assessments have been developed to assess child mental health and functioning, these measures typically have a narrow focus with limited utility. As a result, multiple assessments are often required to evaluate the child or youth’s strengths and needs in various settings and to capture the wide array of features typically associated with the presenting problem (35). This uncoordinated approach to assessment often results in redundancies in information collected, additional resources, and increased assessor burden on the child/youth and their families (35, 36). Critically, previous assessment measures have yet to create a coordinated, cross-sector approach to children’s mental healthcare, or facilitate a lifespan approach to mental health service delivery.

This paper provides an overview of the interRAI child and youth suite of mental health assessment instruments, which was developed in response to the need for an integrated mental health system for young persons. These instruments were designed to facilitate a standardized, comprehensive, and coordinated approach to the delivery of mental health services for infants, children, and youth, ultimately supporting a life course approach to assessment. The paper begins with a discussion of the various factors that must be considered to support a person-centered approach to care. The remainder of the paper describes the design, psychometric properties, and applications of interRAI child/youth mental health assessments using examples related to interpersonal trauma.

### Physical Health and Disability

There is a bi-directional relationship between mental and physical health problems. Particularly, children with chronic health conditions are more likely to have mental health issues, and those with mental health problems are more likely to experience physical health problems (37, 38). For example, a large genomic cohort study revealed young persons with physical health problems (i.e., an autoimmune/inflammatory or central-nervous system condition) were more likely to be diagnosed with a mood or neurodevelopmental disorder (38). Furthermore, children with a serious mental health problem were 13% more likely to have a chronic health condition compared to children with no mental health problem (37). While research suggests that comorbid mental and physical health problems persist into adulthood, some studies have found that interventions targeting mental health issues in childhood improve physical health outcomes (39, 40). Taken altogether, the increase in disability and severity in children and youth with comorbid mental and physical health problems suggests the need for a comprehensive mental health assessment and greater coordination between all service providers, particularly medical and mental health professionals, in order to successfully address this “hidden morbidity” (41, 42).

### Family and Social Relationships

There is also a reciprocal relationship between family dynamics and children’s mental health. Specifically, a child or youth’s mental health issue might play a role in family conflict with caregivers and siblings, potentially contributing to the development of ineffective or inappropriate parenting techniques and detrimental family interactions (43). In turn, high family conflict and poor parenting practices can worsen the child’s mental health issue (44–47).

Parenting practices have a direct impact on attachment in young children. Attachment to a primary caregiver is a key developmental goal in early childhood for survival, safety, and security. However, disruptions in attachment behavioral patterns can have a detrimental impact on the physical and mental health of children (e.g., brain development and long-term functioning) (48–50). Earlier assessment and intervention of attachment issues can significantly reduce the negative sequalae associated with these relationship difficulties and improve overall socio-emotional functioning. Family financial security and socioeconomic status can also have a significant impact on mental health outcomes. For example, a systematic review examining the relationship between socioeconomic inequalities and mental health problems in children and adolescents reported that low parental education and household income had a greater impact on mental health problems compared to parental unemployment or low occupational status (51).

With respect to social relationships, children who struggle in their peer relations often experience mental health issues in adulthood (52). A longitudinal study reported predictive links between early social isolation, poor peer acceptance, and perceptions of social incompetence with subsequent internalizing problems, while early aggression and peer rejection were predictive of subsequent externalizing problems (53–56). Social support can mitigate the negative impact of mental illness as peer connectedness may be a strong protective factor against anxiety, depression, and suicide, thereby bolstering positive self-esteem and general mental well-being (57). Assessment of factors in relation to parenting, family relationships, attachment, financial security, as well as social relations are all crucial in identifying the strategies for effective intervention and treatment. Interactions between social and familial factors often occur simultaneously, creating a more complex approach to address assessment and system needs (58).

### Substance Use

Children and youth with poor family and social relationships are at greater risk for substance use (59, 60). Substance use among young people is a significant public health concern, as it is one of the most commonly cited reasons for admission into a mental health setting (61). The overall prevalence of co-occurring problems in adolescents is just under 3%, with young people between the ages of 15–24 years being more likely to experience substance use disorders (SUD) and/or mental illness compared to any other age group (62, 63). Studies have shown that individuals who suffer from mental health or addiction issues are more likely to die prematurely, with mental illness decreasing a person’s life expectancy by 10–20 years (64).

There are a variety of elements influencing substance use among children and youth, such as peer pressure, trauma, an insecure attachment with caregivers, as well as the presence of a mental health problem (65–68). Youth with both substance use and mental health problems are at risk of serious short- and long-term consequences. For instance, youth with co-occurring disorders are more likely to have impaired functioning, a history of criminal activity, and are typically less responsive to treatment (6, 69–71).

The relationship between substance use and mental illness is a complex one. While numerous theories have been proposed over the years attempting to explain this comorbidity, such as self-medication, the reverse-causal pathway, and shared vulnerability, there is ultimately no simple cause-effect relationship (72). Thus, comprehensive assessment is needed to obtain a better understanding of the interplay between substance use and mental health symptoms, in order to provide effective treatment. Furthermore, many of these youth receive services across multiple service sectors, and so, they require enhanced planning to support transitions across systems of care.

### Transitions

Due to transitions being a lengthy and complex process, it is unsurprising that up to 60% of youth in mental health services lose access to treatment as they transition to adult services (73). Unfortunately, youth who present with a number of complex needs, cultural differences, and/or general distrust of the healthcare system are at greatest risk for ineffective transitions, and therefore, are most likely to experience gaps in their care (74–76).

There are numerous barriers to successful transitions such as the lack of communication between service providers, long wait lists, lack of a common language across sectors, and differing levels of involvement for families in decision-making processes (77, 78). Even small transitions can be difficult for young persons who have difficulty adapting, with particularly challenging transitions across educational placements and service transitions (76). Children and youth who do not have the needed support systems in place often require additional resources to support their care. Because transitions often co-occur with changes in professional relationships, it is quite common for youth to experience feelings of abandonment from their service providers during this time (79, 80). By recognizing these issues and bridging service gaps, positive transitions to support proper care planning can be facilitated, thereby increasing the chances of successful outcomes (76).

### Education

A greater focus on mental health functioning in educational settings may promote learning and prevent the adverse outcomes linked to untreated mental health issues (81). Studies have reported that learning problems predict anxiety, depression, and substance use during adolescence (82–84). In addition, children and youth with mental health concerns are at increased risk of academic struggles (85–87). When children have both mental health and academic problems, they are at greater risk of negative distal outcomes, including a higher likelihood of mental health service utilization, poor academic achievement, special education placement, and school disengagement (88). Notably, over 50% of students (14 years of age or above) who have emotional and behavioral difficulties drop out of high school (89). Overall, mental health problems are significant barriers to learning and school success, and positive behavioral and emotional health are related to academic achievement (90, 91). Therefore, early identification of mental health concerns among students is of significant importance to ensure appropriate access to community resources, effectively providing young persons with a continuum of care.

### Youth Justice

Youth involved in the juvenile justice system experience high rates of mental health problems and trauma (92–94). Approximately 65% of justice-involved youth meet criteria for a mental health disorder, compared to 10–25% of youth in the general population (93). Furthermore, justice-involved youth experience a high level of comorbidity and are 5-times more likely to have one or more mental health disorders compared to the community sample (95).

There is substantive overlap between the mental health needs of youth receiving mental health services and those involved with the justice system (6). Unfortunately, many young persons with mental health concerns are often directed toward the juvenile justice pathway, particularly those with oppositional defiant disorder (ODD), conduct disorder (CD), and substance use issues (96, 97). Ultimately, many justice-involved youth do not receive the mental health care they need – a problem exacerbated by the incarceration of juveniles in adult prisons, which do not provide mental health services designed for youth.

### Traumatic Life Events

Traumatic life events can have debilitating lifelong consequences for children and youth (98, 99). The Adverse Childhood Experiences (ACE) Study examined the impact of family dysfunction and childhood abuse on numerous health outcomes later in life and found that exposure to traumatic events in childhood was strongly associated with mental and physical health problems in adulthood (e.g., depression and heart disease) (100–102).

Traumatic events can be categorized as interpersonal or non-interpersonal in nature (103). Interpersonal traumatic events include those that are directly “human induced [and] involve a malicious perpetrator, one who consciously intends to harm another human being,” (e.g., sexual or physical abuse) (p. 2502) (104). In contrast, non-interpersonal traumatic events lack a malicious perpetrator (e.g., car accidents), or the effects of a malicious perpetrator indirectly impact the individual (e.g., being the victim of crime) (104). Children and youth who have experienced interpersonal trauma are more likely to have internalizing and externalizing problems, such as depression and aggression (105), as well as poor self-image, difficulties in self-regulation, and increased risk of self-harm (106, 107). Overall, interpersonal trauma has a negative effect on psychological, social, and emotional well-being.

Extant research has shown that chronic activation of a child’s stress response system can have an adverse impact on a wide-range of aspects of typical development, such as the development of secure attachments and higher order cognitive skills (108, 109). Notably, many children who experience traumatic events often have a diagnosable mental health disorder, namely Attention Deficit/Hyperactivity Disorder (ADHD), anxiety disorders, ODD, reactive attachment disorder (RAD), and communication disorders (108). Due to the plethora of adverse consequences of traumatic life events, comprehensive assessment is needed to understand how trauma relates to mental health concerns across the lifespan.

## The interRAI Child and Youth Suite of Mental Health Instruments: An Integrated Assessment System

interRAI is a not-for-profit collaborative of over 100 clinicians, researchers, and policy experts from over 35 countries. Although its initial focus in the early 1990s was on geriatrics, the scope of interRAI’s research broadened to include vulnerable persons with complex needs of all ages, including infants, toddlers, and school-aged children.

interRAI’s primary aim is to improve quality of life and care through the development and implementation of an integrated suite of comprehensive assessment, screening, and care planning systems that span the continuum of care. The development of the child/youth suite of instruments in the last decade currently comprises 11 comprehensive assessments, screeners, and supplements (6 finalized, 5 in final stages of refinement). The following section provides a brief description of the child/youth suite using interpersonal trauma as a common clinical theme to show how these instruments provide insights into a young person’s preferences, strengths, and needs.

Before outlining the assessment and screening instruments within the child and youth suite, the paper will define some important key terms. An “infant” refers to an individual from birth to 12 months of age; a “toddler” refers to an individual over 12 months (12 months and 1 day) to 3 years of age; and a “preschooler” refers to an individual 3 years and 1 day to 3 years and 11 months. Furthermore, a “child” refers to an individual over 4 years (4 years and 1 day) to 12 years of age; and a “youth” refers to an individual 12 years and 1 day to 21 years of age.

### Assessment and Screening Instruments for Children and Youth

#### Published Assessment Tools

##### interRAI Child and Youth Mental Health Instrument and Adolescent Supplement

The interRAI Child and Youth Mental Health Instrument (ChYMH) is the primary tool in the child and youth suite of instruments (110). It is used in mental health settings at admission, discharge (if more than 7 days after admission), every 90 days (for longer term patient stays), or when there is a clinically significant change in the young person’s status that potentially requires care plan modifications. The target audience is children and youth between the ages of 4–18 years. The ChYMH is intended to support quality indicators, outcome measurement, case-mix classification, and comprehensive care planning in 30 domains, including social and peer relationships, education, traumatic life events, sleep disturbance, and life skills.

The ChYMH has 31 summary scales (e.g., externalizing and internalizing) and four algorithms (i.e., assessments of harm to self, harm to others, caregiver distress, and resource intensity) embedded within it to support symptom monitoring and care planning. The tool uses specific look-back periods to provide valid and reliable measures of clinical characteristics that represent the young person’s preferences, strengths, and needs. Most items employ a 3-day observation period; however, some items use a 7-day, 30-day, or 90-day window, or lifetime estimates depending on the nature of the issue. Notably, some items address the frequency and/or recency of symptoms prior to and within the last 3 days. The tool also provides the option to indicate that symptoms are present but not exhibited within the last 3 days. An example item from the ChYMH that falls under the ‘Mental State Indicators: Mood Disturbance’ section is “sad, pained, or worried facial expression.” This item can be coded from 0 (not present) to 4 (exhibited daily in last 3 days, 3 or more episodes or continuously). The instrument is typically completed by mental health professionals, such as social workers or nurses, and takes approximately 1 h to complete (dependent on case complexity).

The ChYMH assessment has two versions available, the in-patient and community-based forms (see Table 1). The in-patient version is used for young persons residing in a psychiatric unit/facility or residential facility, whereas the community-based version is used for young persons who reside within the community. Importantly, in the event that a child or youth is transferred from an in-patient to community-based setting, the discharge assessment is shared to support continuity of care. Finally, both versions of the ChYMH include an Adolescent Supplement, which is completed for children and youth who are 12 years of age or above. The supplement can be used for children below 12 years of age if they are engaging in mature behaviors such as substance use or sexual activity.

**TABLE 1:** Item counts by domain area and interRAI mental health system for child/youth populations (ages 0–18).

Characteristic	interRAI Assessment or Screening Instrument*													
	interRAI Early Years	ChYMH Screener	ChYMH Inpatient	ChYMH Community	ChYMH-DD Inpatient	ChYMH-DD Community	EDU	YJCF	ESP-CY	PEDS-HC	QoL-ChYMH	Youth Justice Supplement	Adolescent Supplement	Adolescent Addictions Supplement
Setting	Outpatient and developmental programs	School settings, inpatient and outpatient programs	Inpatient psychiatry	Community (case management)	Inpatient psychiatry	Community (case management)	School settings	Youth justice settings	Emergency department, mobile crisis	Home care	Inpatient and outpatient programs	Youth justice settings	Inpatient and outpatient programs	Addictions programs
Age	0–47 months	4–18 years	4–18 years	4–18 years	4–21 years	4–21 years	4–20 years	12–18 years	4–18 years	4–20 years	7–18 years	12–18 years	12–18 years^4^	12–18 years
***Item counts***														
Administrative and tracking	74	34	49	44	49	46	38	48	43	54	12	20	13	13
Mental status indicators	46	26	46	46	51	51	43	45	37	32	0	3	4	0
Substance use/addictions	3	5	7	7	7	7	6	20	11	5	0	4	11	22
Harm to self/others	0	9	15	15	15	15	14	16	13	5	0	0	0	0
Behavior	30	6	26	24	43	43	32	43	8	17	0	25	6	11
Cognition	31	1	7	7	8	8	8	9	6	11	0	0	0	0
Functional status	112	1	29	29	30	30	11	13	5	68	1	0	13	0
Communication and vision	67	3	6	6	18	18	24	5	0	8	0	0	0	0
Physical health conditions	21	0	26	26	28	28	6	24	1	37	2	0	1	0
Stress and trauma	27	10	32	32	28	28	32	26	3	1	0	0	3	0
Medications	17^1^	1	13^1^	12^1^	13^1^	12^1^	11^1^	11	4	10	0	0	0	0
Service use and treatments	73	0	32	32	40	40	39	31	3	61	4	0	3	7
Control interventions	0	0	6	0	6	1	0	10	0	0	0	0	0	0
Nutritional status	33	0	3	3	4	4	3	7	3	7	0	0	1	0
Social relations	13	2	28	28	28	28	27	21	10	18	13	3	1	2
Employment, education and finances	17	4	26	26	25	25	48	14	2	27	2	0	1	1
Housing, home environment and living arrangements	30	2	20	20	21	20	15	30	6	27	8^3^	5	0	0
Diagnoses	81	2	28^2^	30^2^	29^2^	29^2^	33^2^	32^2^	19^2^	58		0	0	1
Strengths and resilience	0	0	5	5	7	7	5	6	0	0	3	0	1	0
**Total**	675	106	404	392	450	440	395	411	174	446	45	60	58	57

^1^An additional detailed list of medications used in the last 3 days is optional. ^2^Section allows for entry of additional DSM/ICD diagnoses as needed. ^3^The post-service outpatient/inpatient version has an additional item/two items, respectively. ^4^Supplement can be utilized if the child is younger than 12 years of age and engaging in mature behaviors. *The full names of each instrument are not included here due to space, but they can be found within the body of the text.

The ChYMH was piloted in Ontario and has been adopted by 90 children’s agencies within the province. Additionally, several organizations in other provinces (e.g., British Columbia, Newfoundland and Labrador, Quebec, Prince Edward Island, and Nova Scotia) have now implemented the interRAI children’s mental health system. The instrument was published in 2015 and is currently available in both English and Canadian-French. To date, over 20,000 assessments have been completed; notably, this data has been utilized in a number of research studies [e.g., (111–113)].

##### interRAI Child and Youth Mental Health Instrument for Developmental Disabilities and Adolescent Supplement

The interRAI Child and Youth Mental Health Instrument for Developmental Disabilities (ChYMH-DD) is intended to be used with children and youth 4–21 years-old with intellectual or developmental disabilities [e.g., autism, Down syndrome; (114)]. The ChYMH-DD assessment has two versions available; the in-patient and community-based forms, supporting the same broad range of applications as the ChYMH. The instrument supports care plan development in 23 domains, including accessibility and mobility, injurious behavior, continence, educational support, modified nutrition intake, life skills, and social relations.

The ChYMH-DD inpatient and community-based forms are typically completed by developmental service workers. The tool mirrors the ChYMH with respect to the scales and algorithms embedded within it, its specific observation periods, and approximate time for completion. Further, the ChYMH-DD similarly has an Adolescent Supplement. The ChYMH-DD was piloted within Ontario and Finland and is now standard of care in Newfoundland and Labrador, Prince Edward Island, and Nova Scotia. The instrument was published in 2015 and is currently available in both English and Canadian-French. To date, just over 1,000 assessments have been completed, with several interRAI papers examining this particularly vulnerable population [e.g., (115–117)].

##### interRAI Child and Youth Mental Health Screener

The interRAI Child and Youth Mental Health Screener (ChYMH-S) is a brief standardized assessment tool that is intended to complement the full ChYMH and ChYMH-DD assessments (118). The ChYMH-S was designed to support decision-making related to triaging, placement, and service urgency for young persons with mental health issues. This basic screening tool has the same target audience as the previously described instruments, namely children and youth between the ages of 4–18 years. Further, the tool can be used in various settings, such as in-patient and out-patient programs and educational environments.

The ChYMH-S is comprised of items largely selected from the ChYMH instrument, with some additional items specific for screening purposes. The tool has similar look-back periods to that of the ChYMH and ChYMH-DD and takes approximately 15–20 min to complete by trained clinical staff. It is important to note that while the ChYMH-S informs immediate care triaging, it is not an alternative to or a replacement for the full ChYMH or ChYMH-DD; more pointedly, it is intended to identify those young persons who are in need of a more comprehensive mental health assessment. Furthermore, the ChYMH-S does not support care plan development. Lastly, the tool has three clinical scales and three algorithms (i.e., assessments of harm to self, harm to others, and service urgency) embedded within it. The algorithm related to service urgency is called the Children’s Algorithm for Mental Health and Psychiatric Services (ChAMhPs). ChAMhPs provides a score ranging from 0–6, with higher scores indicative of more urgent and emergent cases. A score of three or higher indicates a full ChYMH should be completed based on case complexity.

The ChYMH-S was initially piloted within Ontario and China. The tool was published shortly after the comprehensive ChYMH and ChYMH-DD assessments in 2017 and is now used as part of standard of care across most mental health agencies in the Province of Ontario. It is currently available in English, Canadian-French, and Simplified Chinese. Over 80,000 assessments have been completed thus far, with a number of research studies utilizing the ChYMH-S data [e.g., (119–121)].

##### interRAI Pediatric Home Care

The interRAI Pediatric Home Care Assessment (PEDS-HC) is a clinical tool intended for children and youth between 4–20 years of age who receive home-based medical care due to their complex health needs (122). The instrument is predominantly used to support decision-making with respect to the allocation of funding for children and youth who are medically complex and require home-based services. It supports several wide-ranging applications (e.g., quality indicators and outcome measurement), and employs similar specific observation periods.

The PEDS-HC is comprised of over 400 items with scales, algorithms, and care plans in development. The PEDS-HC was developed in the United States and has been piloted within Ontario. It is currently used in Ontario, Nebraska, Texas, and Maryland. The tool was published in 2014, is currently available in English, and almost 500 assessments have been completed to date.

##### Self-Reported Quality of Life – Child and Youth Mental Health

The interRAI Self-Reported Quality of Life – Child and Youth Mental Health (QoL-ChYMH) is a self-report survey that assesses the perception of well-being and life satisfaction of young persons 7–18 years of age prior to and after receiving mental health services (123). The tool fosters child and youth engagement and involvement in treatment planning and goal setting, thus providing young persons with an opportunity to impact service delivery. The purpose of the QoL-ChYMH is to identify areas of strengths and needs of young clients, with the ultimate goal of maximizing quality of life.

The QoL-ChYMH is comprised of four major domains, which are then further subdivided into 10 categories based on protective factors and indicators of positive mental health that are well-established in the literature: (1) Basic Needs (living conditions, food, safety, and privacy), (2) Social (friends and activities, respect from others, family), (3) Individual (autonomy, health), and (4) Services (school, treatment). Three harmonized versions of the tool have been developed, including pre-service for in-patient and out-patient programs, post-service for in-patient programs, and post-service for out-patient programs. The QoL-ChYMH can be used in conjunction with the ChYMH and Family Quality of Life tool to gain insight into the young person’s perspective and inform treatment planning. The tool takes around 15 min to complete. Approximately 1,000 assessments have been completed to date, and research studies have begun to utilize the QoL-ChYMH data [e.g., (124)]. Similar to the other instruments, the QoL-ChYMH was piloted in Ontario and has been recommended as best practice by Accreditation Canada.

##### Family Quality of Life – Child and Youth Mental Health

The interRAI Family Quality of Life – Child and Youth Mental Health (FamQoL) is a survey that assesses the perception of the family’s well-being and life satisfaction prior to and after their child receives mental health services (123). Similar to the self-report tool, the FamQoL assists with the promotion of family engagement by involving an adult family member (preferably the primary caregiver) in the child or youth’s treatment planning. The purpose of the FamQoL is to help service providers identify areas of strengths and difficulties from the family’s perspective, in order to maximize treatment outcomes and quality of life of both the child/youth and their family.

Similar to the QoL-ChYMH, the FamQoL was developed in collaboration with expert clinicians well-versed in family dynamics within the context of children’s mental health, and in line with well-established protective factors and indicators of positive mental health. The FamQoL tool consists of seven domains, namely Safety, Informal Support, Formal Support, Community Interaction and Leisure, Family Relationships and Interactions, Life Circumstances, and Interpersonal Challenges. Two versions of the tool have been developed, including the pre-service and post-service for in-patient/out-patient programs. Importantly, the FamQoL can be used in combination with the ChYMH and QoL-ChYMH to gain insight into the family’s perspective on strengths/needs as well as service satisfaction. This QoL tool takes approximately 10–15 min to complete. The FamQoL was piloted in Ontario and approximately 1,200 assessments have been completed thus far.

#### Assessments at Pilot Stage

##### interRAI Youth Justice Custodial Facilities

The interRAI Youth Justice Custodial Facilities (YJCF) is a comprehensive standardized instrument for youth between the ages of 12–18 years who currently reside in custodial facility settings^2^. The YJCF has several wide-ranging applications, including quality indicators, resource allocation, and comprehensive care planning in 26 domains (e.g., criminality prevention, sexual offending, harm to others, self-harm, family functioning). It is available in both English and Canadian-French.

The YJCF utilizes a standard 3-day look back period across several areas. A 3-year Ontario pilot study from 2015–2018 yielded 90 completed assessments and was used to provide descriptive profiles of youth in custody compared to those in mental health settings (6, 70).

##### interRAI Early Years

The interRAI Early Years is a comprehensive standardized tool that is intended to be used with infants, toddlers, and children between 0–47 months who are demonstrating developmental, emotional, social, or behavioral concerns^3^. The tool has a similar breadth of scope regarding its applications, with a key one being comprehensive care planning. The 17 care planning protocols triggered by the instrument address issues related to attachment, caregiver distress, nutritional intake, traumatic life events, gross/fine motor, and toilet training readiness. Further, the tool currently has three scales and algorithms, with others in development.

The interRAI Early Years assesses five developmental milestones, namely cognition, socio-emotional development, expressive and receptive language, gross motor, and fine motor skills. The tool has similar specific observation periods as the other instruments previously described. The interRAI Early Years is also designed to be directly compatible with other interRAI assessments for young persons such as the ChYMH and ChYMH-DD. The synchronization among the tools within the child/youth suite sets the foundation for a seamless transition between services for infants, toddlers, and school-aged children (4–18 years). Importantly, service providers can monitor a child’s progress through consistent outcome measurement from infancy to adulthood. The interRAI Early Years was piloted over a 3-year period, from 2016–2019, within Ontario. Notably, over 1,000 assessments have been completed thus far with publication anticipated by 2022.

##### interRAI Child and Youth Emergency Screener for Psychiatry

The interRAI Child and Youth Emergency Screener for Psychiatry (ESP-CY) evaluates the needs of young persons with mental health issues who present to crisis or emergency services, such as psychiatric emergency departments, general emergency departments, or mobile crisis teams^4^. Like the ChYMH, the ESP-CY is typically completed by mental health professionals, such as nurses and social workers. Designed to inform decision-making related to patient safety, placement, and service utilization, the ESP-CY can also provide important information and valuable insights at the beginning of an in-patient mental health episode.

The ESP-CY is based on, and complements, the full mental health assessments (i.e., ChYMH and ChYMH-DD). Considering the ESP-CY has a different clinical focus, namely an emphasis on patient safety and acute symptoms, the basic time frame is set to the last 24 h unless otherwise indicated. Consequently, some items address the frequency and recency of symptoms prior to and within the last 24 h.

The target audience of the ESP-CY is young persons between 4–18 years-old. The tool can be used in a variety of settings, including both in-patient and community-based services and programs. The average time for completion is 15–20 min, although it can vary depending on the acute nature of the child or youth’s symptoms and availability of other informants, such as family members. The ESP-CY has two care planning protocols and three basic risk appraisal algorithms that assess risk of harm to self, risk of harm to others, and inability to care for self. Importantly, while the instrument informs immediate safety planning, it is not an alternative to or replacement for the full ChYMH or ChYMH-DD assessments. The ESP-CY is now being piloted in Ontario.

##### interRAI Education

The interRAI Education (EDU) is intended to be used with young persons referred to school-based psychological or mental health services^5^. The tool has the same target audience as the PEDS-HC, which is children and youth between 4–20 years of age. It is designed to support a similar broad range of applications, including case-mix classification, quality indicators, outcome measurement, and comprehensive care planning. Particularly, the tool supports care plan development in 30 domains, such as communication, attention and learning supports, vision and hearing impairment, strengths, classroom management/discipline, and is intended to support school engagement (129–131).

The interRAI EDU essentially provides an assessment of key domains of function, educational needs, mental and physical health, and social support. Certain items on the tool can also identify those students who are at higher risk for specific problems related to well-being, health, and functioning, and may require further evaluation. Importantly, the EDU is compatible with other instruments in the interRAI child/youth suite. The tool also mirrors the ChYMH and ChYMH-DD with respect to specific observation periods (i.e., default set to 3-days), and its Adolescent Supplement. Similar to the interRAI ChYMH and ChYMH-DD, there are several decision-support algorithms within the instrument.

##### interRAI Adolescent Addictions Supplement

The interRAI Adolescent Addictions Supplement is an ancillary clinical tool that is intended to be used with young persons who struggle with addictive behaviors^6^. The supplement is designed for use in conjunction with the ChYMH and ChYMH-DD. Specific scales and care planning protocols for this supplement are in development. Youth can fall into four different addictive behavior streams to support intervention and treatment planning (i.e., video gaming, gambling, tobacco use, and substance use). The supplement employs the same basic time frame (3 days) unless otherwise indicated.

The Adolescent Addictions Supplement should always be completed for young persons who are receiving treatment for addictive behaviors. When triggered, it is strongly advised to complete it shortly after the full mental health assessment (i.e., preferably the same day). When the supplement is not triggered, it can still be completed based on clinician discretion. A number of items are taken into account when deciding whether the supplement should be completed, including “smokes tobacco daily,” “number of days in last 30 days consumed alcohol to point of intoxication,” “gambled excessively or uncontrollably,” “problem video gaming in last 90 days,” among others.

### Data Holdings Utilized to Demonstrate interRAI Applications

Before outlining the factors that make the child/youth suite an integrated system, it is important to provide an overview of the interRAI data holdings that will be used to illustrate concepts in this and subsequent sections. Analytic data for the results presented are drawn from assessments collected in Ontario mental health settings. The interRAI Early Years assessment is used as part of regular clinical assessment practice in 15 mental health agencies serving very young children from October 2016 to August 2020. The interRAI ChYMH-S, ChYMH, and ChYMH-DD are used by 62, 59, and 13 agencies, respectively, as part of regular clinical assessment practice. The ChYMH and ChYMH-DD are drawn from assessments done between September 2012 and August 2020, while ChYMH-S records were completed between April 2014 and August 2020. The YJCF data is drawn from a pilot implementation in 9 agencies between November 2014 and August 2015. Finally, OMHRS is a mandated implementation of the interRAI-MH in all hospital-based adult in-patient psychiatry units. All residents with stays of 72 h or longer are to receive a comprehensive admission assessment, and these were used if the patient was 21 years of age or younger at the time of the assessment. For these six instruments, an encrypted individual level identifier was available to select the first assessment of individuals if there was more than one. Of note, for the baseline and follow-up treatment used in one set of outcomes, pairs of assessments for individuals were selected between 30 and 365 days apart. Please see the Appendix for additional information regarding data holdings.

### An Integrated Health Information System

There are a number of key features that make the child/youth suite of instruments an integrated health information system. All these instruments have a primary clinical focus on comprehensive functional assessment of strengths and needs to support care planning and outcome measurement across diverse groups [e.g., (133)]. The intended clinical use is not for diagnosis; rather, the instruments capture existing medical and psychiatric diagnoses. All instruments also have a common conceptual emphasis on care planning protocols. These collaborative action plans (CAPs) use empirically derived triggering algorithms to flag areas of potential need and provide evidence-informed guidelines for engaging youth and their support network in a shared decision-making approach while incorporating individual strengths, preferences, and needs (see text foonote 1, 134).

This integrated system also provides a common language with consistent terminology to define common concepts across all settings (e.g., mental health, youth justice, and education), as well as transitions throughout the lifespan (e.g., from infancy to adulthood), thereby improving the effectiveness of communication. Importantly, items only differ between the child/youth and adult suite when it makes sense to do so from a developmental perspective. Also, some items appear in some, but not all, instruments because they are relevant only in specific developmental stages.

The interRAI system uses standardized data collection methods with a detailed training manual that includes intent, definitions, and coding rules for each item. Assessors typically complete a 2.5-day training for the comprehensive interRAI Child and Youth instruments (e.g., the ChYMH, ChYMH-DD, and interRAI Early Years) and a full day training for the briefer instruments (e.g., ChYMH-S). The trained child/youth mental health professionals include psychologists, nurses, psychiatrists, speech and language therapists, child and youth workers, developmental social service workers, and social workers. All available sources of information are utilized to complete the assessment (i.e., family members, community members, document review, and clinical observations). Utilizing multiple forms of information, the assessments do not use fixed narrative questions, but rather employ a semi-structured interview format, thereby providing assessors flexibility in how data is gathered. The interRAI child/youth suite also has a set of core items shared across tools that allow for population-level analyses of issues that are pertinent to children’s mental health.

### Psychometric Properties of interRAI Child and Youth Mental Health Instruments

One of the many benefits of adopting research-based mental health instruments for decision-making when providing services to children, youth and their families is the ability to consistently and accurately measure the constructs of interest. Reliability and validity are two psychometric properties that should be considered upon administration of an instrument and interpretation of the collected data (135). The interRAI child and youth mental health instruments and their associated elements, such as scales and algorithms, have gone through extensive reliability and validity testing to ensure their use across multiple service sectors [e.g., (12, 20, 112, 113, 120, 136–145)].

#### Reliability

##### Inter-Rater Reliability

It is imperative that assessments conducted by different trained clinicians result in consistent outcomes. Inter-rater reliability or inter-observer agreement is the consistency of results taken from assessments across trained clinicians (135). interRAI assessments have undergone rigorous inter-rater reliability testing with both children and adults (34, 146). One approach to obtain inter-rater reliability strictly utilizes vignettes, which can result in inflated values; however, interRAI takes a more rigorous approach, in that it is conducted as part of typical clinical practice (34). This is exemplified in the obtainment of inter-rater reliability for our newest instrument, the interRAI Early Years. Here, assessors familiar with the tool independently conducted a review of the case file, collateral contacts, and related information while conducting an assessment. Further, they documented their findings with the young child and their family. Results indicated strong precision between raters with values of ICC = 0.98 ([95% CI, 0.97, 0.99], *p* < 0.001) for expressive and receptive language scales and ICC = 0.87 ([95% CI, 0.72, 0.94], *p* < 0.001) for the gross motor scale (146). Similarly, several items within the child and youth instruments are shared across the entire interRAI suite, which have demonstrated strong inter-rater reliability [see (141, 144, 147)].

##### Item Reliability or Internal Consistency

Another form of reliability is item reliability or internal consistency, which is the extent that items in a single assessment measure the same construct (135). Item reliability is routinely measured by Cronbach’s alpha (135, 148). Within the interRAI suite, this form of reliability has been used to evaluate new scales; moreover, it has also been used to help with quality assurance of the data obtained from widespread implementation (34). Numerous studies have been conducted examining the internal consistency of scales and algorithms derived from the interRAI child and youth suite of instruments (138–141, 144, 146). Table 2 describes several clinical summary scales that are available in the child/youth suite, and Table 3 summarizes the internal consistency results for symptoms and behaviors of interest across various instruments. Almost all the scales meet or exceed an alpha level of 0.70 or 0.80 indicating fair or good/moderately high reliability, respectively (135).

**TABLE 2:** Summary of scales and algorithms in interRAI child/youth mental health instruments.

interRAI scale	Domain	Type of scale	Scale components	Range	Included in
Activities of Daily Living Scale	Basic physical function	Parallel form Sum of items	Bathing; Personal hygiene; Dressing upper body; Dressing lower body	0–8	ChYMH, ChYMH-DD, EDU, YJCF
Anxiety Scale	Frequency and intensity of anxiety symptoms	Parallel form Sum of items	Repetitive anxious concerns; Unrealistic fears; Obsessive thoughts; Intrusive thoughts or flashbacks; Episodes of panic; Hypervigilance; Nightmares	0–28	ChYMH, ChYMH-DD, ChYMH-S, EDU, YJCF
Autism Spectrum Screening Checklist (ASSC)	Frequency and intensity of symptoms related to autism	Parallel form Sum of items	Narrowly restricted range of interest; Excessive preoccupation with activity or routine; Lack of social/emotional conventions when socializing; Excessive or unusual reaction to sensory stimuli; Difficulty adapting to even minor change	0–5	ChYMH, ChYMH-DD, ChYMH-S interRAI Early Years, EDU, YJCF
Caregiver Distress Algorithm (iCCareD)	Degree and diversity of caregiver distress factors	Decision Tree	Proactive aggression; Reactive aggression; Parenting strengths scale; Disruptive/aggressive behavior scale; Aggressive behavior scale; Family functioning composite score	1–5	ChYMH, ChYMH-DD, EDU, YJCF
Children’s Algorithm for Mental Health and Psychiatric Services (ChaMhPS)	Level of urgency and need for a full comprehensive assessment	Decision tree	Intent to quit school; Intrusive thoughts; Nightmares; Hyperactivity; Lack of interest in social interaction; Lack of motivation; Negative statements; Guilt or shame; Being socially inappropriate; Risk of family/placement breakdown; Considered self-injury; Others concerned about self-injury; Violence to others; Danger to self; Danger to others; Being a victim of emotional abuse	0–6	ChYMH-S Please note: A score of 3+ on the ChaMhPS indicates a full assessment is required due to case complexity and need for individualized care planning.
Child and Youth Resource Index (ChYRI)	Case-mix classification system to inform resource allocation	Decision tree	Age; Supportive relationship with family; Fine motor skills; Violence to others; Bladder continence; History of foster family placement; Maternal substance use during pregnancy	3-to-1 range	ChYMH-DD, EDU, YJCF
Communication Scale	Level of expressive and receptive communication	Parallel form Sum of items	Making self-understood; Ability to understand others	0–8	ChYMH, ChYMH-DD, EDU, YJCF
Disruptive/Aggressive Behavior Scale	Frequency and severity of aggressive and disruptive behavior	Parallel form Sum of items	Verbal abuse; Physical abuse; Socially inappropriate/disruptive; Destructive behavior toward property; Outbursts of anger	0–20	ChYMH, ChYMH-DD, EDU, YJCF, ESP-CY
Depressive Severity Index	Severity of depressive indicators	Parallel form Sum of items	Sad facial expressions; Negative statements; Self-deprecation; Guilt or shame; Hopelessness	0–15	ChYMH, ChYMH-DD, ChYMH-S, EDU, YJCF, ESP-CY
Externalizing Scale	Frequency of externalizing symptoms (i.e., behavioral disturbance); consists of 2 factors- proactive aggression and reactive aggression	Parallel form Sum of items	*Proactive Aggression Factor:* Stealing; Elopement attempts/threats; Bullying peers; Preoccupation of violence; Violence to others; Intimidation of others or threatened violence; Violent ideation *Reactive Aggression Factor:* Impulsivity; Physical abuse; Outburst of anger; Defiant behavior; Argumentativeness	0–12	ChYMH, ChYMH-DD, EDU, YJCF
Hyperactive/Distraction Scale	Frequency of hyperactivity and distractibility behaviors	Parallel form Sum of items	Impulsivity; Distractibility; Hyperactivity; Disorganization	0–16	ChYMH, ChYMH-DD, ChYMH-S, EDU, YJCF, ESP-CY
Instrumental Activities of Daily Living (Capacity and Performance)	Higher level physical functioning	Parallel form Sum of items	Ordinary housework; Phone use; Use of technology; School tasks; Orientation in familiar environment; Stairs; Meal preparation; Managing finances; Managing medications; Shopping; Transportation	0–66	ChYMH, ChYMH-DD, EDU, YJCF
Internalizing Scale	Frequency and severity of internalizing symptoms; consists of 3 factors- anhedonia, anxiety, and depression	Parallel form Sum of items	*Anxiety Factor:* Repetitive anxious complaints/concerns; Hypervigilance; Unrealistic fears; Episodes of Panic *Anhedonia Factor:* Lack of motivation; Anhedonia; Withdrawal from activities of interest; Decreased energy *Depression Factor:* Made negative statements; Self-deprecation; Expressions of guilt/shame; Expressions of hopelessness	0–48	ChYMH, ChYMH-DD, EDU, YJCF
Pain Scale	Frequency and intensity of pain	Parallel form Sum of items	Pain frequency; Pain intensity	0–4	ChYMH, ChYMH-DD, EDU, YJCF
Parenting Strengths Scale	Degree of strengths that the parent is demonstrating in parenting activities	Parallel form Sum of items	Communicate effectively with child/youth; Assists child/youth with the regulation of emotions; Uses appropriate disciplinary practices; Demonstrates warmth and support; Appropriate supervision and monitoring; Appropriate limit setting or expectations	0–12	ChYMH, ChYMH-DD, interRAI Early Years, EDU, YJCF
Peer Conflict Scale	Level of conflict with friends	Parallel form Sum of items	Conflict with or repeated criticism of close friends; Friends are persistently hostile or critical of child/youth; Pervasive conflict with peers (exclude close friends)	0–3	ChYMH, ChYMH-DD, EDU, YJCF
Positive Symptoms Scale	Frequency of positive symptoms of psychosis	Parallel form Sum of items	Hallucinations; Command hallucinations; Delusions; Abnormal though process	0–12	ChYMH, ChYMH-DD, ChYMH-S, EDU, YJCF, ESP-CY
Resource Intensity for Children and Youth Algorithm (RIChY)	Intensity and nature of service needs	Decision tree	Intimidation of others or threatened violence; Destructive behavior toward property; Conflict/repeated criticism of close friends; Friends persistently hostile; Bullying peers or conflict with peers; Family Functioning Scale; Victim of crime; Victim of sexual assault; Victim of physical assault; Victim of bullying; Victim of emotional abuse; Witnessed domestic violence; Constipation; Seizures; Dry mouth; Hypersalivation; Dyspnea; Akathisia; Dyskinesia; Tremor; Bradykinesia; Rigidity; Dystonia; Slow shuffling gait; Other emergent conditions; Parenting Strengths Scale; Difficulty falling asleep; Anxiety Scale; Self-Harm CAP; Harm to Others CAP	0–5	ChYMH, ChYMH-DD, EDU, YJCF
Risk of Injury to Others (RIO)	Injury to others	Decision tree	Physical abuse; Violence to others; Threatened violence; Violent ideation; Destructive behavior; Verbal abuse; Socially inappropriate behavior; Impulsivity; Family overwhelmed	0–6	ChYMH, ChYMH-DD, EDU, YJCF
Risk of Suicide and Self Harm in Kids (RiSsK)	Suicide and self-harm	Decision tree	Intent to kill self; Considered self-injury; Attempted self-injury; Others concerned about self-injury; Self-injurious behavior; Family overwhelmed; Depression Symptoms Scale	0–6	ChYMH, ChYMH-DD, EDU, YJCF
School Disengagement Scale	Intensity of school disengagement	Parallel form Sum of items	Increase in lateness or absenteeism; Poor productivity or disruptiveness at school; Expresses intent to quit school; Conflict with school staff; Strong, persistent dissatisfaction with school; Child/youth refuses to attend school; Child/youth removed due to disruptive behavior; Overall academic performance	0–8	ChYMH, ChYMH-DD, EDU, YJCF
Social Disengagement Scale	Frequency of symptoms related to anhedonia	Parallel form Sum of items	Anhedonia; Withdrawal from activities of interest; Lack of motivation; Lack of interest in social interaction	0–16	ChYMH, ChYMH-DD, ChYMH-S, EDU, YJCF, ESP-CY
Sleep Difficulties Scale	Frequency of symptoms related to sleep difficulties	Parallel form Sum of items	Difficulty falling asleep or staying asleep; Wakes multiple times at night; Falls asleep during the day (exclude naptime); Resists bedtime	0–16	ChYMH, ChYMH-DD, EDU, YJCF

**TABLE 3:** Internal consistency of clinical scales derived from interRAI child/youth mental health instruments.

	Instrument				
	ChYMH-S	ChYMH	ChYMH-DD	YJCF	OMHRS < 21
*N*	*81,207*	*20,935*	*1,042*	*90*	*36,244*
Depressive Severity Index (0–15; 5 items)	0.800	0.812	0.779	0.790	0.736
Anxiety Scale (0–28; 7 items)	0.732	0.761	0.684	0.848	n/a
Disruptive/Aggressive Behavior Scale (0–20; 5 items)	0.831	0.851	0.779	0.817	n/a
Hyperactive/Distraction Scale (0–16; 4 items)	0.803	0.805	0.671	0.813	n/a
Parenting Strengths Scale (0–12; 6 items)	n/a	0.888	0.877	n/a	n/a
Externalizing Scale (short form) (0–14; 7 items)	0.739	0.781	0.683	0.736	n/a
Internalizing Scale (short form) (0–44; 11 items)	0.822	0.834	0.795	0.846	n/a

#### Validity

##### Face and Content Validity

A reliable instrument is necessary, but not sufficient, to prompt the adoption of an assessment system for clinical practice. Validity demonstrates that the assessment measures what it intends to capture, with face or content validity referring to the extent to which the assessment’s items represent the construct being investigated (135). During the development of each assessment tool, interRAI establishes face or content validity through consultation with clinicians and researchers, as well as a thorough review of the current literature (34, 144). For example, as part of the developmental efforts of the internalizing and externalizing scales for the interRAI ChYMH, a panel of experts in the field were tasked with evaluating the content representativeness. These efforts resulted in scales with excellent content validity [e.g., (112, 137)].

The interRAI ChYMH consists of over 400 clinically relevant items that have shown strong face validity in evaluating a child or youth’s strengths, needs, and functioning related to presenting psychiatric, medical, and social issues (110). Strong face and content validity have been demonstrated throughout the test development phase with extensive international feedback. Notably, many items from the ChYMH are shared across other child and youth instruments (i.e., ChYMH-S, ChYMH-DD, YJCF, EDU, PEDS-HC, and Early Years). Additionally, certain items from the child/youth suite are similar to those found in the adult suite to aid with the sharing of information between clinicians in different service sectors and across age groups (34). Such an approach fosters continuity of care across service sectors as well as transitions into adult services. All of the interRAI scales, algorithms, and associated features of the assessment systems are created in a similar, rigorous manner.

##### Construct Validity

Once the construct has been defined and key items have been selected for inclusion in the assessment tool, it is important to test whether the items are associated in a way that would be expected. There are two forms of construct validity, namely convergent and discriminant validity. Convergent validity examines whether similar factors of a construct are shown to be related to each other, whereas discriminant validity highlights when two dissimilar items are shown to be unrelated (148). Items within the child/youth suite of instruments have demonstrated strong construct validity (112, 136, 138, 139, 141–143).

Lau and colleagues (136) assessed the ability of the Disruptive/Aggressive Behavior Scale (DABS) and Hyperactive/Distraction Scale (HDS) to discriminate between two groups that are known to differ on the elements of interest. This was completed through the comparison of mean scores of DABS and HDS for children and youth with and without disorders of interest based on the Diagnostic and Statistical Manual of Mental Disorders – Fourth Edition (DSM-IV) criteria. Furthermore, comparisons were made between the values of the area under the curves. Results suggested that DABS could differentiate between disruptive behavior disorders (DBD) and ADHD. Similarly, HDS was able to differentiate between an ADHD diagnosis relative to a DBD diagnosis with area under the curve values of over 0.70, indicating good discrimination for the measures. These results coincide with our previous work that found scores on the Social Disengagement Scale, which measures the frequency of symptoms related to anhedonia, were strongly associated with a mood disorder diagnosis (143). Similarly, the Depressive Severity Index (DSI) has been found to be highly related to a mood disorder diagnosis, while the Anxiety Scale has been found to be related to anxiety disorders in children and youth with and without intellectual disabilities (138, 142).

With respect to Activities of Daily Living (ADLs), Stewart and colleagues (113) developed and validated two ADL summary scales for both children and youth with normative intellect and developmental disabilities; both scales have shown strong psychometric properties. Results from this study highlighted that no clear hierarchical structure was observed, particularly when stratified by age. However, children and youth with developmental disabilities exhibited a higher level of dependence in daily tasks. Furthermore, both ADL summary scales had excellent internal consistency. A study conducted by Phillips and colleagues (139) investigated the psychometric properties of two scales measuring the activity limitations of a non-clinical sample of children with chronic illnesses. The results of the study supported discriminant validity with Pearson’s *r* = −0.0174 (*p* = 0.46) and −0.025 (*p* = 0.28) for the PEDS-HC Activities of Daily Living Limitations Scale and the Hands-on Needs Scale, respectively.

##### Criterion-Related Validity

Criterion-related validity compares the scores from an assessment against a particular outcome; two types include concurrent and predictive validity (135). Concurrent validity demonstrates the correlation between the scores under investigation with scores belonging to another assessment that evaluates the same construct (139). In contrast, predictive validity is based on correlations of scores on one assessment with scores on a criterion measure obtained in the future (135, 148).

###### Concurrent Validity

Numerous studies suggest that the tools in the child/youth suite have strong concurrent validity (112, 137, 138, 141, 142). A common approach to this type of validity is to compare the assessment tool under investigation with a tool that is recognized as a “gold standard” for the construct of interest (34). For example, Lau and colleagues (112, 137) found that the internalizing and externalizing subscales from the interRAI ChYMH had strong concurrent validity by comparing them with relevant criterion measures from other validated assessment instruments: Beck Youth Inventories, Social Skills Improvement System (SSIS), Child and Adolescent Functional Assessment Scale (CAFAS), Child Behavior Checklist (CBCL), and the Brief Child and Family Phone Interview (BCFPI). The strongest correlations were exhibited with the SSIS internalizing and externalizing behaviors and CBCL internalizing and externalizing scales, respectively, as resultant Pearson’s Rho Bayesian Correlations were greater than 0.60. Additionally, Stewart and Babcock (141) conducted a similar study examining scales on the ChYMH-S (i.e., anxiety, distractibility/hyperactivity, internalizing, and externalizing). Their findings suggested that the ChYMH-S scales were significantly and positively correlated with relevant criterion scales from The Behavior Assessment System for Children, Third Edition (BASC-3). More recently, Li and colleagues (148) examined the Depressive Severity Index against the CBCL’s Internalizing Scale and the BASC-3’s Depression Scale and found evidence supporting the DSI’s concurrent validity with a resultant Pearson’s correlation of *r* = 0.450, *p* = < 0.001 and *r* = 0.613, *p* < 0.001, respectively.

###### Predictive Validity

One of the most difficult forms of validity to establish is predictive validity (34). A number of studies have shown that various scales within the interRAI child and youth suite have strong predictive validity (138, 139, 143). For example, data from over 5,000 children and youth placed in adult psychiatric settings in Ontario suggested that the Aggressive Behavior Scale was predictive of multiple control intervention use, such as use of restraints. For each unit increase on the Aggressive Behavior Scale, there was a 54%, 62%, and 34% increase in the odds of having received a mechanical restraint, physical restraint, or been held in seclusion, respectively (143). The Severity of Self-Harm (SOS) scale has been useful in predicting admission for risk of self-harm in youth between the ages of 10–17 years, as well as suicide in inpatient settings several years later. Specifically, Hirdes found that individuals who were considered to be high-risk on the SOS were 6.82-times more likely to die by suicide (unpublished data). Similarly, the DSI has been found to significantly predict self-injurious attempts and suicide intent (138). This form of validity is extremely important as one of the main goals of clinical work is to have a positive influence on the child or youth’s developmental trajectory (34). The child and youth Caregiver Distress algorithm was recently developed and validated to identify factors associated with, and predictive of, new or ongoing distress among caregivers referred for mental health services (149). Utilizing longitudinal data, it was found to predict new or ongoing caregiver distress in parents of treatment-seeking children and youth, providing valuable clinical information to prevent future family breakdown. Additionally, the Risk of Injury to Others (RIO) is a decision-support tool developed and validated in order to identify children and youth at increased risk for violence (150). Findings indicated that it was a strong predictor of harmful behavior toward others, and it also predicted increased likelihood of future aggressive behaviors. These decision-support algorithms can be utilized to support strategic prevention and early intervention efforts for these vulnerable youth to circumvent negative sequelae when many of these features present early in life.

Table 4 also highlights the predictive validity of a selected set of scales within the suite. Here, it is evident that an increase in odds ratios for provisional psychiatric diagnoses is related to higher scale values; this means that individuals who scored higher on a particular scale were more likely to have a provisional diagnosis of the associated disorder. For example, those who scored higher on the Hyperactive/Distraction scale were more likely to have a provisional diagnosis of ADHD. Furthermore, goodness of fit ranged from 0.71 to 0.83 after adjusting for age group and sex, suggesting good/moderately high predictive validity.

**TABLE 4:** Odds ratios (95% CL) for provisional psychiatric diagnoses by associated symptoms.

Provisional Diagnosis	*Prevalence*	Explanatory measure/scale	Adjusted^1^ OR (95% CI)	c-stat
Attention deficit hyperactivity	*43.3%*	Hyperactive/Distraction Scale: 0	ref	0.780
		1 to 2	2.85 (2.37–3.43)	
		3 to 9	7.72 (6.58–9.06)	
		10 to 16	21.15 (17.83–25.07)	
Anxiety	*43.0%*	Anxiety Scale: 0 to 2	ref	0.716
		3 to 7	2.29 (2.10–2.49)	
		8 to 12	3.33 (2.96–3.75)	
		13 to 28	4.83 (4.10–5.70)	
Mood	*21.2%*	Depressive Severity Index: 0	ref	0.791
		1 to 3	1.45 (1.26–1.67)	
		4 to 7	1.97 (1.70–2.28)	
		8 to 15	2.71 (2.31–3.19)	
		Social Disengagement Scale: 0	Ref	
		1 or 2	1.20 (1.06–1.37)	
		3 to 8	1.57 (1.39–1.77)	
		9 to 16	2.04 (1.70–2.43)	
Learning or communication	*19.9%*	Communication Scale: 0	ref	0.726
		1 to 2	2.41 (2.17–2.68)	
		3 to 4	3.34 (2.86–3.89)	
		5 to 8	4.49 (3.30–6.10)	
		Daily decision making: impaired	2.03 (1.84–2.25)	
Disruptive behavior disorder	*18.8%*	Disruptive/Aggressive Behavior Scale: 0	ref	0.725
		1 to 3	3.16 (2.66–3.76)	
		4 to 9	7.33 (6.21–8.64)	
		10 to 20	14.08 (11.65–17.03)	
Autism spectrum	*9.6%*	Autism Spectrum Screening Checklist: 0	ref	0.827
		1	4.13 (3.36–5.08)	
		2	8.91 (7.19–11.04)	
		3	15.34 (12.30–19.12)	
		4	32.45 (25.78–40.85)	
		5	46.82 (35.25–62.18)	

*N = 13,951. ^1^Adjusted for age group and sex.*

### Use and Utility of the interRAI Child and Youth Suite

Some of the key fundamental measures that are available in all or most of the six instruments previously described in the section outlining our data holdings are summarized in Table 5. Considering the different target ages and clinical needs that these instruments are designed for, the ability to measure and report across populations and in a valid and comparable way is a key feature of this family of instruments. For example, about 1 in 10 individuals assessed with the interRAI Early Years instrument report a family member feeling overwhelmed, and this is approximately 2 in 3 among those assessed with the YJCF or ChYMH-DD instrument. This table also highlights that fewer than 10% of those children/youth assessed with the ChYMH, ChYMH-DD, or ChYMH-S had a lifetime suicide attempt, whereas this number jumps to 22% of those assessed in youth justice and 30% of those assessed in inpatient psychiatry. Experiencing abuse is notably higher in the youth justice sample, particularly physical and emotional abuse. Furthermore, younger persons assessed in inpatient psychiatry had the highest prevalence of sexual abuse, at nearly 1 in 5.

**TABLE 5:** Summary of fundamental measures available across child/youth instruments.

	Instrument					
	interRAI Early Years	ChYMH-S	ChYMH	ChYMH-DD	YJCF	OMHRS (up to 21)
*N*	*1,106*	*81,207*	*20,935*	*1,042*	*90*	*36,244*
Mean age (std. dev)	2.3 (1.0)	12.4 (3.8)	12.4 (3.6)	12.1 (3.6)	17.2 (0.8)	19.3 (1.8)
Male	65.1%	49.1%	54.6%	72.1%	79.2%	52.9%
Lives with parent/guardian	92.6%	92.0%	90.2%	83.1%	63.9%	n/a
History of foster placement	8.7%	n/a	13.4%	18.0%	33.3%	n/a
Custody dispute	5.2%	n/a	5.0%	1.9%	n/a	n/a
Any lifetime residential/inpatient admission	n/a	n/a	18.5%	21.8%	29.2%	33.7%
Children’s Aid Society (CAS) is legal guardian	3.8%	1.7%	3.8%	6.7%	0.0%	n/a
Family report feeling overwhelmed	10.6%	35.7%	44.5%	64.8%	57.9%	44.0%
Victim of bullying (ever)	n/a	42.2%	47.7%	24.9%	55.6%	n/a
Witnessed domestic violence	7.6%	23.2%	28.3%	19.9%	44.4%	n/a
Any self-injurious attempt to kill self	n/a	8.6%	9.5%	3.9%	22.2%	30.8%
Violent ideation in last year	n/a	6.5%	11.0%	10.8%	43.1%	15.6%
Sexual abuse	0.4%	8.7%	11.1%	5.6%	16.7%	19.3%
Physical abuse	1.1%	13.5%	18.9%	15.5%	51.4%	28.0%
Emotional abuse	2.5%	22.7%	27.7%	17.1%	55.6%	18.6%
Any of 3 trauma items (physical, sexual, emotional)	3.0%	29.2%	34.9%	22.6%	68.1%	37.1%

**FIGURE 1:**
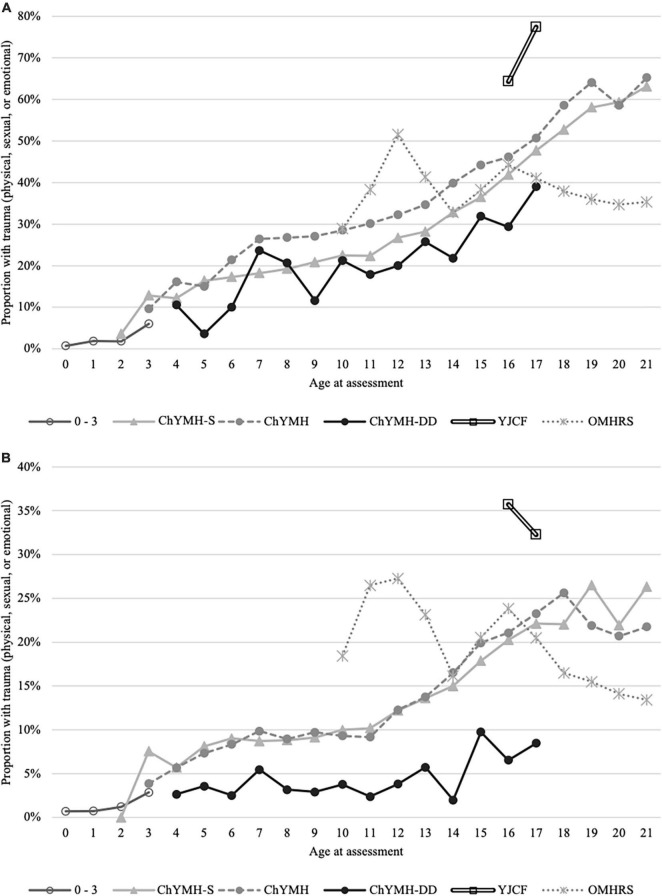
**(A)** Lifetime trauma: victim of sexual assault or abuse, physical assault or abuse, or emotional abuse at any time. **(B)** Trauma in the last year: victim of sexual assault or abuse, physical assault or abuse, or emotional abuse within the last year.

Figure 1A shows lifetime interpersonal trauma, which uses three items that record if the child or youth has been a victim of sexual assault or abuse, physical assault or abuse, or emotional abuse at any time. Figure 1B shows any interpersonal trauma in the last year, to illustrate the extent to which it is not merely the accumulation of more years of exposure that produces increased prevalence with age. As such, both Figures 1A,B show proportions of interpersonal trauma are low among younger ages, and then it generally increases by year. This is evident regardless of which instrument is used, with the exception of younger adults who are assessed with the interRAI-MH as part of the OMHRS system for inpatient psychiatry beds. Here, it is interesting to note that the proportion with interpersonal trauma declines around 17 years of age. Both figure panels also show that young persons assessed with the ChYMH-DD tend to have lower levels of interpersonal trauma, whereas those assessed with the YJCF have exceptionally high levels. It is possible that, due to low communication or verbal skills, children with intellectual disabilities are less likely to disclose such interpersonal trauma. Furthermore, children in regular inpatient units age 13 and younger show more interpersonal trauma, and this is even more apparent when considering the last year only. Finally, Figure 1A highlights that the large number of individuals assessed with the ChYMH-S show lower levels of interpersonal trauma compared to those assessed with the more comprehensive ChYMH; however, this pattern is not similarly seen in Figure 1B.

### Applications of interRAI Instruments

All instruments within the child/youth suite are intended to be used as part of standard clinical practice and they each serve a number of different functions. Given the high prevalence of interpersonal trauma within children’s mental health (i.e., approximately 40% depending on the interRAI instrument used), this construct was utilized to illustrate the differences in applications, specifically across the variety of scales/algorithms, care planning protocols, outcome measurement, and quality indicators.

#### Care Planning

A unique feature of interRAI assessments is that they integrate a comprehensive evaluation of an individual’s areas of strengths and needs within a series of collaborative action plans. Within each instrument, these care planning guidelines have been developed to inform clinical decision-making. Each CAP is comprised of the same five elements: (1) a description of the clinical issue, (2) goals of care, (3) an overview of the various triggering levels, which are based on certain items from the assessment and are associated with different approaches to care, (4) guidelines to assist with care planning based on international best practice, and (5) additional resources related to that particular clinical issue, including references to peer-reviewed publications.

As noted, specific items within each assessment tool serve as “triggers” for certain CAPs, which are subsequently used to help provide evidence-informed care. It is important to emphasize that the purpose of CAPs is to support needs-based care planning; hence, they are not prescriptive in nature. In addition, it is essential that CAPs are utilized within a client-centered approach to care. This means that they are used to help facilitate shared decision-making. More specifically, the child/youth and their family are an integral part of the discussion with regard to how the main areas of need identified by the CAPs will be addressed moving forward. These CAPs have been extensively reviewed by over 150 experts in their respective fields across at least three continents.

Table 6 shows 12 selected CAPs across the ChYMH, ChYMH-DD, and YJCF. The ChYMH cases show the highest triggering rates for medication adherence, whereas the ChYMH-DD cases show the highest triggering rates for communication, medication review, and strengths. Furthermore, YJCF cases show the highest triggering rates for substance use, hazardous fire risk, self-harm, harm to others, interpersonal conflict, transitions, and traumatic life events. Finally, cases across all three instruments show similar triggering rates for sleep disturbance, which is approximately 40% of assessed individuals.

**TABLE 6:** List of selected CAPs and their triggering rates across three child/youth mental health instruments.

	Instrument		
CAP	ChYMH (*n* = 20,887)	ChYMH-DD (*n* = 1,042)	YJCF (*n* = 90)
Self-harm/suicide	24.4%	12.3%	41.7%
Harm to others	12.3%	1.9%	25.0%
Traumatic life events	51.0%	38.1%	61.1%
Interpersonal conflict	54.9%	47.5%	65.3%
Transitions	22.7%	41.8%	72.2%
Substance use	17.9%	1.4%	87.5%
Medication adherence	12.0%	8.9%	6.9%
Medication review	21.7%	40.4%	11.1%
Hazardous fire involvement	4.6%	4.7%	8.7%
Communication	11.8%	65.6%	15.3%
Sleep disturbance	39.9%	42.6%	40.3%
Strengths	16.9%	40.6%	13.9%

**FIGURE 2:**
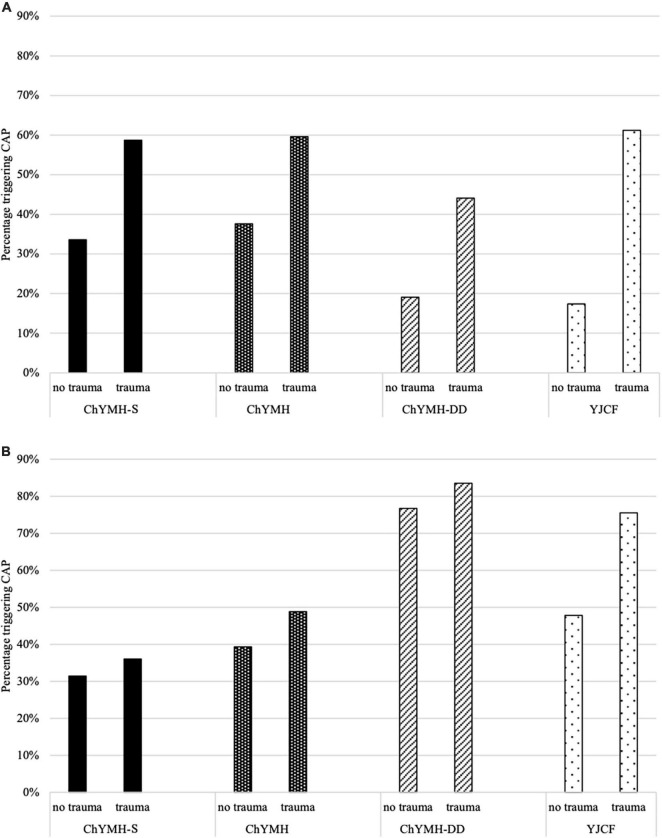
**(A)** Triggering rates for the Suicidality and Purposeful Self-Harm CAP, by trauma and instrument. **(B)** Triggering rates for the Harm to Others CAP, by trauma and instrument.

Figure 2A shows the triggering rates for the Suicidality and Purposeful Self-Harm CAP by interpersonal trauma and instrument. It is quite evident that a greater proportion of those who have experienced interpersonal trauma trigger the Suicidality and Purposeful Self-Harm CAP, regardless of instrument/cohort. This link between interpersonal trauma and increased risk of suicidality and self-harm is well-supported by the literature [e.g., (151–153)]. Figure 2B shows the triggering rates for the Harm to Others CAP by interpersonal trauma and instrument. These results show a similar pattern as the Suicidality and Purposeful Self-Harm CAP, whereby a greater proportion of those with a history of interpersonal trauma trigger the Harm to Others CAP across instruments/cohorts. Previous research has also found a relationship between these traumatic experiences and risk of harm to others [e.g., (154–156)].

#### Outcome Measurement

Scales embedded within interRAI instruments help capture the complexity of the areas of need at a given point in time. These scales provide a clinical summary of an individual’s status across key domains (e.g., cognitive functioning, depression) and are automatically calculated upon completion of the assessment. Across all instruments, higher scores indicate greater symptom severity, problems related to functioning, or frequency of occurrences. Overall, interRAI scales are useful in describing the individual’s current level of functioning and supporting care planning; furthermore, when examined longitudinally, they provide insights about response to intervention and changes in strengths and needs over time.

**FIGURE 3:**
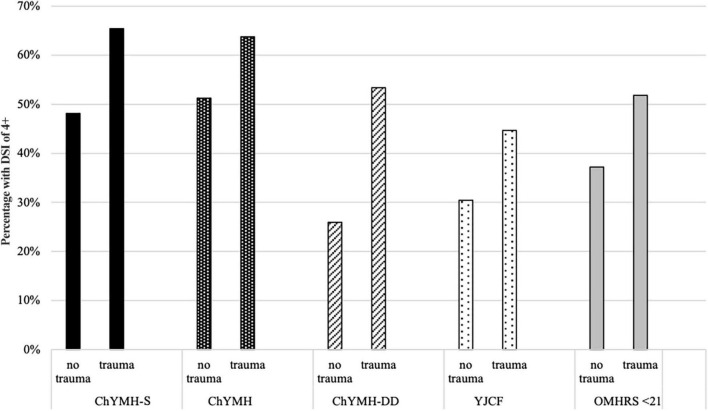
Percentage distributions of Depressive Severity Index score of 4 or greater, by trauma and instrument.

Scales can be utilized to highlight differences in mental health severity across in-patient and out-patient services, and to examine outcomes to determine treatment effectiveness (157). An example within the child/youth suite is the Depressive Severity Index, which sums 5 items to produce a scale from 0 to 15, where higher values indicate greater depressive symptoms. In Figure 3, the DSI outcome scale is used to show higher depressive symptoms among those with a lifetime history of interpersonal trauma, compared to others without such a history, across 5 instruments used in child/youth mental health populations.

#### Quality Indicators

Quality indicators (QIs) are summary measures that can provide a comprehensive understanding of quality of care. While outcome scales can be used to track changes in strengths and needs over time at the individual level, longitudinal data can be compiled to track performance at the population level. QIs serve multiple functions; for example, they can be used by agencies to monitor care and facilitate internal quality improvement. Furthermore, QIs can be used to assist the government with accountability and public awareness. These indicators often look at two main outcomes, namely improvement in symptoms or failure to improve/worsening of symptoms between admission and follow-up.

**FIGURE 4:**
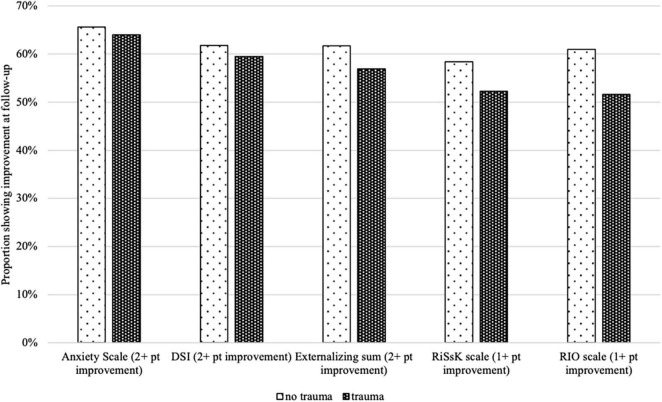
Change from initial to follow-up assessment, by scale and trauma.

Figure 4 depicts the proportion of children and youth showing improvement at follow-up across five selected scales, by interpersonal trauma status. Based on the findings, the presence of interpersonal trauma history negatively impacts recovery. Specifically, it is less likely for measures of anxiety, depressive symptoms, externalizing symptoms, risk of self-harm and injury to others to improve at follow-up, compared to those without a history of interpersonal trauma. Furthermore, it is important to note that baseline rates are higher in those who have experienced trauma, which means they have a greater opportunity to improve by at least the threshold points, and yet they do not.

The interRAI child/youth suite is currently in the process of further developing QIs to reflect changes in patterns of symptom levels and domains of functioning. A more thorough examination of the relationship between individual characteristics and differential outcomes will help determine which sub-populations respond well to certain interventions, compared to those that do not. As a result, this will help facilitate differential triaging and, in turn, effective resource allocation. Finally, it can also help identify areas that would benefit from the development of innovative approaches to intervention, including novel approaches to trauma-informed care.

#### Resource Allocation

interRAI assessment systems can also be used to inform decisions with respect to resource allocation at the individual and societal level. For example, within the adult suite of instruments, the interRAI-MH instrument was used to develop a level of care framework to support resource allocation. Notably, such a framework or decision support algorithm had not previously been available for pediatric populations. Specifically, within the children’s mental health setting, decisions about resource allocation were often based on unstandardized instruments and subjective interpretations, thereby reducing the likelihood that resources were allocated fairly and effectively. In response to this need, interRAI launched an effort to develop a decision support algorithm for resource allocation among younger populations and created the Resource Intensity for Children and Youth (RIChY) (20).

Resource Intensity for Children and Youth is an empirically based decision-support tool that may be used to inform the nature and intensity of scope of service needs for children and youth needing facility- or community-based services. RIChY is based on item responses on full comprehensive assessments. The RIChY algorithm divides children and youth into three age groups: 7 and under, 8 to 11, and 12 and older. The algorithm provides a score ranging from 0–5 based on levels of need for intensive resources; however, not all ages populate all categories. Specifically, the algorithm ranges from 0–3 for children 7 and under, whereas it ranges from 0–5 for children 8 to 11 years and those 12 and older. Higher scores on the algorithm indicate a greater priority for intensive services.

The child or youth can fall into a given level via a number of pathways that represent different combinations of risk factors, such as intimidation of others or threatened violence, external circumstances (e.g., traumatic life events, family dysfunction, or lack of close friends), and risk of harm to self or others. While the algorithm is a decision-support tool, it is important to emphasize that it is ultimately the responsibility of the clinical team to use professional judgment to decide if the RIChY score accurately reflects the young person’s need for intensive services, given all available information.

**FIGURE 5:**
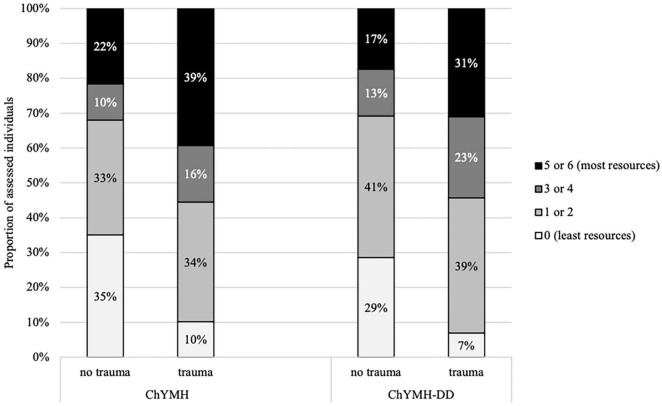
Distribution of the Resource Intensity for Children and Youth (RIChY) scale.

Figure 5 shows the distribution of the RIChY scale by interpersonal trauma status across the ChYMH and ChYMH-DD. For both ChYMH and ChYMH-DD cases, expected resource intensity is higher in those with a history of interpersonal trauma compared to those without such a history (i.e., there is a greater proportion in high resource groups, and a lower proportion in low resource groups).

**FIGURE 6:**
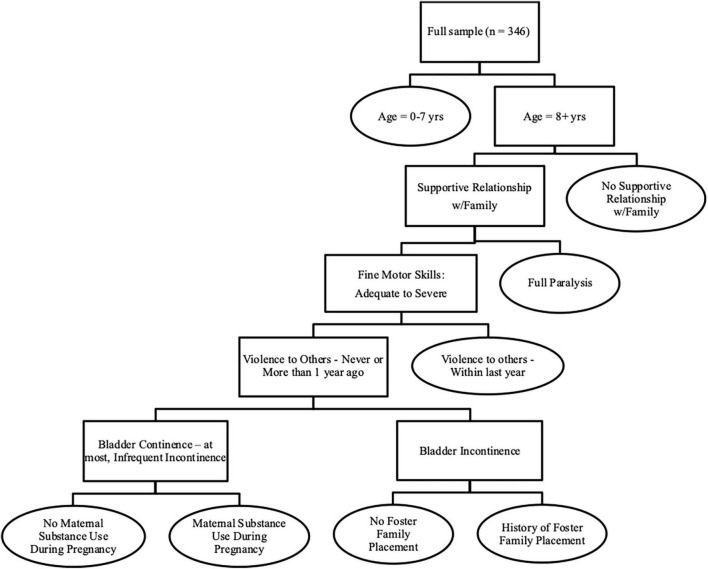
The interRAI Child and Youth Resource Index (ChYRI). Ovals represent terminal ChYRI groups.

Another tool that can be used to inform decisions regarding the allocation of resources within the field of healthcare are case-mix classification systems. Case-mix modeling utilizes information about individual characteristics in conjunction with resource data to create groupings based on similar resource requirements. Hence, case-mix classification systems typically describe the comparative resource needs of different groups, with payment systems then attaching a dollar value to these various case-mix groups. Within the adult mental health sector, a case-mix system was developed and implemented across the Province of Ontario, namely the System for Classification of In-Patient Psychiatry (SCIPP) (34). Unfortunately, most service sectors typically utilize funding models based on a standard rate per client rather than the complexity of their needs. To address this gap within the children’s mental health sector, the Arkansas Division of Developmental Disability Services (ARDDS) collaborated with interRAI to develop a case-mix classification system to inform decisions regarding the allocation of resources for children and youth with intellectual and developmental disabilities (IDD). This effort was part of a system-wide payment reform initiative for Medicaid. As a result of this effort, the interRAI Child and Youth Resource Index (ChYRI) was created (shown in Figure 6).

Child and Youth Resource Index is an empirically based decision-support tool that can be used to inform resource allocation among young persons with IDD (158). It serves as a useful guideline to aid in decision-making around allocation of resources and planning for appropriate services. The classification system incorporates 8 distinct groups and explains 30% of the variance in *per diem* costs. A number of variables are included within the system, such as age, motor skills, violence to others, supportive relationship with the family, foster placement, and certain health conditions (e.g., paralysis, bladder continence). There is a 3-to-1 range in case-mix indexes (CMIs) across the groups. Overall, ChYRI can be utilized to improve equitability in the allocation of limited resources within vulnerable populations, centered around stability and fairness.

## Future Directions

Over the past decade, the child and youth mental health suite of instruments has made significant contributions within both clinical and research domains. Nevertheless, in order to continue to make a meaningful impact, some of the limitations of the suite need to be addressed. For the suite’s potential to be fully realized, it is important for the leaders of mental health teams to emphasize the clinical utility of the system rather than the aspects related to data collection. It is also important to emphasize the value of effective communication and collaboration across various service sectors and healthcare providers in order to maximize the utility of this integrated system. Additionally, ensuring there is a strong, well-designed software system to support data collection and uptake of the scales, algorithms, and care plans to ensure proper use and utility of the interRAI suite is key.

Notably, the interRAI instruments exemplify a needs-based approach to care, which supports a more equitable approach to service delivery, and consequently, reduces disparities in the provision of mental health services (159). This approach will only become increasingly vital as the social inequities around the world continue to rise, and we are tasked with navigating new global challenges, as evidenced by the COVID-19 pandemic [see (160–162)]. Therefore, the interRAI integrated assessment system is both well-equipped and well-positioned to help inform equitable service delivery to ensure that those who are most vulnerable and in greatest need have increased access to services and receive the appropriate resources in an efficient, effective manner.

The implementation of the interRAI instruments has been highly effective in Ontario with implementation across other provinces currently underway. Future research is needed across Canada as well at the international level. It will be essential to continue to build partnerships with health leaders from different countries, including those from low-, middle-, and high-income nations. Furthermore, the development of additional self-report tools is presently underway, which will be especially useful in serving parts of the world where there are a lack of mental health professionals and significant barriers to accessing mental health services. These instruments will also support new implementation efforts within primary care.

While many studies have already been published examining the validity and reliability of the child/youth mental health instruments, additional research in this area is needed including international studies investigating the areas of predictive validity, criterion validity, and inter-rater reliability. It will also be important to consider developing case-mix systems for in-patient and community mental health instruments for children and youth in general, as the ChYRI is only intended for those with IDD.

Finally, as the child/youth suite of mental health instruments continues to grow and develop, it will create a plethora of new and exciting opportunities that will be transformative in nature. For example, these longitudinal datasets comprised of rich clinical information will create opportunities for applying artificial intelligence tools, thereby expanding possibilities related to the development of novel applications for personalized health care systems. Most critically, a comprehensive mental health assessment system that spans from infancy to adulthood will provide an extraordinary opportunity to examine the impact of mental health and illness on all ages and stages, and develop innovative solutions to help each individual maximize their quality of life.

## Author Contributions

SS and JP developed the analytical strategy. JP performed the statistical analysis. All authors contributed to the formulation of the ideas presented in the study and were involved in the writing and reviewing of the final manuscript.

## Conflict of Interest

The authors declare that the research was conducted in the absence of any commercial or financial relationships that could be construed as a potential conflict of interest.

## Publisher’s Note

All claims expressed in this article are solely those of the authors and do not necessarily represent those of their affiliated organizations, or those of the publisher, the editors and the reviewers. Any product that may be evaluated in this article, or claim that may be made by its manufacturer, is not guaranteed or endorsed by the publisher.

## Footnotes

1 Stewart SL, Toohey A. <arttitle>Screening and assessment of mental health problems in students: utilizing an integrated assessment-to-intervention approach to enhance integrated care. In: Andrews J, Shaw S, Domene J, McMorris C editors. *School, Clinical, and Counseling Psychology: Mental Health Assessment, Prevention, and Intervention*. New York, NY: Springer (under review).

2 Stewart SL, Leschied AW, Hirdes J, Mathias K, Currie M, McKnight M, et al. *interRAI Youth Justice Custodial Facilities (YJCF) Assessment Form and User’s Manual*. Washington, DC: interRAI (in pilot).

3 Stewart SL, Iantosca JM, Klassen JA, Cloutier S, Tucker M, Fisman S, et al. *interRAI Early Years Assessment Form and User’s Manual. Version 10.0*. Washington, DC: interRAI (in pilot).

4 Stewart SL, Hirdes J, Morris JN, Berg K, Björkgren M, Declercq A, et al. *interRAI Emergency Screener in Psychiatry for Child and Youth (ESP-CY) Assessment Form and User’s Manual. Version 1*. Washington, DC: interRAI (in pilot).

5 Stewart SL, McKnight M, Currie M, Gilpin M, Evans B, Richards P, et al. *interRAI Education (EDU) Assessment Form and User’s Manual. Version 1*. Washington, DC: interRAI (in pilot).

6 Stewart SL, Perlman C, Hirdes J, Curtin-Telegdi N, Berger J, Ferris J, et al. *interRAI Adolescent Addictions Supplement to the Child and Youth Mental Health (ChYMH), Child and Youth Mental Health-Developmental Disabilities (ChYMH-DD) and Child and Youth Emergency Screener for Psychiatry (CY-ESP). Version 10*. Washington, DC: interRAI (in pilot).

## Appendix

**Supplemental Data Description**

All data used were from extracts of assessments captured by secure software platforms used by the assessing agencies. If an individual was assessed more than once by an agency using the same instrument, the first observation was used.

Data description, by instrument:

**Table:**

Instrument	Start Date	End Date	*N*	Lifetime Trauma		Number of Agencies
				No	Yes	
ChYMH	Oct 2012	Aug 2020	20,887	13,600 (65.1%)	7,287 (34.9%)	59
ChYMH-DD	Oct 2012	Aug 2020	1,042	806 (77.4%)	236 (22.7%)	13
ChYMH-S	Jan 2015	Aug 2020	81,152	57,471 (70.8%)	23,681 (29.2%)	62
Early Years	Feb 2017	Aug 2020	1,106	1,073 (97.0%)	33 (3.0%)	15
OMHRS (up to age 21)	Oct 2005	Mar 2020	35,705	22,468 (62.9%)	13,237 (37.1%)	90
YJCF	Nov 2014	Aug 2015	72	23 (31.9%)	49 (68.1%)	9

For Figure 4, individuals with two consecutive assessments (ChYMH or ChYMH-DD or ChYMH-Screener) between 30 and 365 days apart were identified. Assessment setting was required to be the same on both assessments, i.e., inpatient for both or outpatient for both. There were 11,552 pairs: 5,791 ChYMH, 124 ChYMH-DD, and 5,637 ChYMH-Screener. We report the proportion of these pairs that achieved a 2-point improvement (Anxiety, DSI, and Externalizing scales) or a 1-point improvement (RiSsK and RIO scales) among cases that had a baseline scale score of 2 more, or 1 or more, respectively. Note that higher scale values reflect higher acuity, so an improvement is a decline in score value.

These N’s were:

Anxiety Scale: 8,176 (3,376 ineligible).

DSI: 8,853 (2,699 ineligible).

Externalizing Scale: 8,661 (2,891 ineligible).

RiSsK Scale: 5,749 (5,803 ineligible).

RIO Scale: 5,992 (5,560 ineligible).
